# Mpox and pregnancy: A call for inclusion of pregnant patients in future research

**DOI:** 10.1016/j.crwh.2023.e00510

**Published:** 2023-05-03

**Authors:** Emily B. Rosenfeld, Todd Rosen

**Affiliations:** Division of Maternal-Fetal Medicine, Department of Obstetrics, Gynecology, and Reproductive Sciences, Rutgers Robert Wood Johnson Medical School, New Brunswick, NJ, United States of America

**Keywords:** Mpox, Infectious disease, Pregnancy

Mpox (formally known as monkeypox) was a rare viral disease in the United States but is endemic to Central and West Africa. Mpox is a zoonotic viral infection that results in a rash similar to smallpox and was declared by the World Health Organization as a public health emergency in July 2022. Mpox is the name recommended by the World Health Organization, which has changed the name from “monkeypox” to avoid the stigma associated with naming diseases after animals or locations. While the disease is typically mild in humans, it can cause severe complications in pregnant persons, including miscarriage and intrauterine fetal demise. Little is known about the disease course during pregnancy and the mechanism for fetal demise. This article aims to highlight the importance of improving our understanding of mpox infection in pregnancy, to develop effective strategies for prevention and management.

The mpox virus is a double-stranded DNA virus that is part of the *Orthopoxvirus* genus of viruses in the Poxvirdae family. Mpox virus has two subtypes: Clade I, which was previously referred to as Congo Basin, is known to cause more severe illness; and Clade II, previously referred to as West African, is currently the predominant strand in the United States. Mpox can be transmitted by direct contact, prolonged exposure to respiratory secretions, or contact with contaminated items or surfaces [1]. As of March 15, 2023, mpox virus has been reported in all 50 states for a composite of 30,262 cases and 38 deaths [2].

The mpox initial infection typically presents with fever, malaise, lymphadenopathy, and myalgia followed by a rash that presents 1 to 4 days the after initial presentation of symptoms. Rashes are usually vesicular or pustular and first appear in the genital, anal, or oral regions. Hospitalizations have occurred in 13% of patients and the incidence of death has been 1 in 796 infections [1,2]. Mpox virus can cause complications like kidney injury, ulcerative pharyngitis, or myocarditis. Infection is diagnosed by an *Orthopoxvirus* polymerase chain reaction. If *Orthopoxvirus* is found on polymerase chain reaction, a mpox virus specific testing is performed for confirmation [1].

The pathogenesis of mpox is not well understood, particularly in the context of pregnancy. The mechanism by which mpox penetrates the maternal-placental-fetal barrier is poorly understood. However, the limited literature on mpox infection during pregnancy has highlighted devastating consequences in some cases. A recent meta-analysis by D'Antonio et al. evaluated the outcomes of 7 cases of mpox infection during pregnancy. These cases were reported from 1983 to 2018 and were limited to African countries where mpox virus is endemic. They reported that 62% had vertical transmission. Miscarriage occurred in 67% of first-trimester infections and 82% of second-trimester infections resulted in an intrauterine fetal demise. In this meta-analysis there were no reports of maternal deaths [3]. There is a single case of mpox infection during pregnancy published during the current outbreak, which resulted in the birth of a healthy neonate [4].

Characteristic ultrasound findings have not been established. However, similar to other viruses in the *Orthopoxvirus* genus, mpox is known to be associated with fetal anomalies such as hepatomegaly and hydrops. Neonates have been born with congenital mpox infection and stillborn fetuses have also been reported to have the characteristic skin findings with presence of mpox infection identified in tissue [1].

Although most mpox infections are mild, about 5% require some sort of treatment. Antivirals and vaccinia immune globulin are available but there is limited data on their effectiveness. The Centers for Disease Control and Prevention advocates that pregnant, lactating and recently pregnant people are prioritized in obtaining treatment for an infection. However, there is no human data on the safety profile of these drugs during organogenesis, pregnancy, or lactation.

There are two vaccines available. The first is ACAM2000, which is a replicating viral vaccine historically used for the prevention of smallpox. It has been linked with pregnancy loss and congenital defects so is contraindicated during pregnancy. JYNNEOS is a nonreplicating viral vaccine approved for both smallpox and mpox disease. Animal studies have been reassuring in pregnancy and the Society of Maternal Fetal Medicined advocates that it is not withheld from pregnant people. However, due to the limited supply, it is currently only available to those with known or suspected exposure to mpox [1]. However, increasing vaccine hesitancy in the United States and worldwide could negatively influence strategies to reduce the burden of disease, even when the vaccine becomes more widely available.

The Centers for Disease Control and Prevention advocates for early use of tecovirimat (also known as TPOXX or ST-246) as first-line treatment for pregnant or recently pregnant patients with a confirmed mpox infection [5]. Tecovirimat is an antiviral medication that is approved by the Food and Drug Association for treatment of smallpox. Pregnant subjects were excluded from the original pharmacokinetic studies of tecovirimat. In one recent publication of tecovirimat treatment during the third trimester, the neonate was born healthy without complications [4]. In addition, no adverse events were observed in supratherapeutic doses given in animal studies. It is unknown if treatment with tecovirimat will prevent congenital mpox infection. Tecovirimat's size makes it likely to cross the placenta and future research is needed to establish the effectiveness of tecovirimat in preventing congenital mpox infections [4].

Mpox infection in pregnancy represents an important area for future research. Similar to many novel infectious diseases over the past century, outcomes may be worse for pregnant people and their fetuses compared to their non-pregnant counterparts. Yet, pregnant subjects are nearly universally excluded from research on vaccinations and treatments of novel infections. Mpox infection is another instance where pregnant patients have been left out of scientific and medical advances. The scientific community should reconsider its exclusion of pregnant patients from research with mpox. Given that pregnancies can end in a fetal demise, the potential benefits may outweigh the theoretical risks of experimental treatments.

## References

[1] Society for Maternal-Fetal Medicine (SMFM) (2022). https://www.smfm.org/mpox.

[2] 2022 U.S. Map & Case Count https://www.cdc.gov/poxvirus/mpox/response/2022/us-map.html.

[3] D’Antonio F., Pagani G., Buca D., Khalil A. (2023). Monkeypox infection in pregnancy: a systematic review and metaanalysis. Am. J. Obstet. Gynecol. MFM..

[4] Sampson M.M., Magee G., Schrader E.A., Dantuluri K.L., Bukhari A., Passaretti C. (2023). Mpox (Monkeypox) infection during pregnancy. Obstet. Gynecol..

[5] Clinical Considerations for Mpox in People Who are Pregnant or Breastfeeding. https://www.cdc.gov/poxvirus/mpox/clinicians/pregnancy.html.

